# Side‐Specific Mastication Prevalence and Its Association With Sex, Anxiety, and Somatic Symptoms: A Cross‐Sectional Study Among Italian University Students

**DOI:** 10.1002/cre2.70348

**Published:** 2026-04-26

**Authors:** Bachar Reda, Luca Contardo, Mariam Hmeidan

**Affiliations:** ^1^ Department of Medical, Surgical and Health Sciences School of Dentistry University of Trieste Trieste Italy; ^2^ Institut National de Santé Publique, d'Epidémiologie Clinique et de Toxicologie‐Liban (INSPECT‐LB) Beirut Lebanon

**Keywords:** Generalized Anxiety Disorder, Patient Health Questionnaire, somatic symptoms, unilateral chewing behavior

## Abstract

**Objective:**

To assess the prevalence of unilateral chewing among Italian university students and investigate its association with sex, anxiety, and somatic symptom severity.

**Material and Methods:**

In this cross‐sectional study, 1536 students completed a self‐administered questionnaire. Prevalence of unilateral chewing behavior, anxiety, and somatic symptoms was described using frequencies and percentages. Anxiety and somatic symptoms were measured using the self‐reported Generalized Anxiety Disorder‐7 (GAD‐7) and Patient Health Questionnaire‐15 (PHQ‐15). Pearson Chi‐square and Cramér's V examined the association between sex and unilateral chewing behavior. Differences in GAD‐7 and PHQ‐15 scores across unilateral chewing categories were analyzed using ANOVA‐based methods with post hoc tests. Ordinal logistic regression was performed to assess the association between unilateral chewing behavior, anxiety, and somatic symptom severity while controlling for age and sex.

**Results:**

Overall, 19% of participants reported unilateral chewing “most” or “all of the time.” Moderate‐to‐severe anxiety and somatic symptoms were each reported by 45% of participants, assessed separately. A significant weak to moderate association between sex and unilateral chewing was reported (*p* < 0.001). Unilateral chewing behavior was significantly associated with total anxiety scores (*p* < 0.001), with “all of the time” chewers having four points higher scores than “none of the time” or “a little of the time,” and three points higher scores than “some of the time” chewers. A significant association (*p* < 0.001) was also observed between unilateral chewing and PHQ‐15 scores, with “all of the time” chewers having three points higher scores than “none of the time” and two points higher scores than “a little of the time.” Ordinal regression analysis confirmed these associations, although the effect sizes were modest.

**Conclusion:**

Frequent unilateral chewing behavior is associated with higher levels of anxiety and somatic symptoms. Assessing unilateral chewing may help guide interventions promoting bilateral mastication and mindful eating.

**Abbreviations**
COVID‐19: Coronavirus Disease 2019
DC/TMD: Diagnostic Criteria for Temporomandibular Disorders
GAD‐7: Generalized Anxiety Disorder‐7
HAM‐A: Hamilton Anxiety Rating Scale
OBC‐21: Oral Behavior Checklist‐21
OHRQoL: Oral Health‐Related Quality of Life
PHQ‐15: Patient Health Questionnaire‐15
PSS: Perceived Stress Scale
SD: standard deviation
TMD: Temporomandibular Disorder

## 1 Introduction

Oral health is fundamental for maintaining proper functioning of the organ systems of the orofacial region, with a direct impact on both physiological and psychological well‐being (Martino 2011; Peres et al. 2019). Maintaining oral health has been linked with the overall oral health‐related quality of life (OHRQoL) according to a population study in China, where individuals with deleterious oral behaviors reported lower OHRQoL scores (An et al. 2022).

The state of an individual's oral health is influenced by various behaviors, some of which are critical for determining oral health status. These behaviors can be categorized into two broad groups: physiological behaviors, including mastication, communication, swallowing, and breathing, and non‐physiological behaviors, such as unilateral chewing, grinding, and clenching (Okada et al. 2007; van der Bilt et al. 2006; Khawaja et al. 2015; Markiewicz et al. 2006; van der Meulen et al. 2014).

Oral behaviors refer to masticatory muscle activities that occur outside the normal physiological behaviors. Research has suggested that frequent engagement in such behaviors is considered a risk factor for a range of dental and psychological conditions, including temporomandibular disorders (TMD), jaw function limitation, anxiety, and depression (Keela et al. 2024; Barbosa et al. 2021; Donnarumma et al. 2021; Michelotti et al. 2025; Xu et al. 2021; Zhong et al. 2024). One of the tools used to assess these behaviors is the Oral Behavior Checklist (OBC‐21), a self‐report scale designed to identify and quantify the frequency of engagement in oral behaviors (Markiewicz et al. 2006; Ohrbach et al. 2008). The OBC‐21 has been validated and is widely used as an integral part of the Diagnostic Criteria for Temporomandibular Disorders (DC/TMD) (Schiffman et al. 2014). This tool has been used in several studies assessing the relationship between oral behaviors and TMD, as well as studies examining the prevalence of oral behaviors among various populations (Keela et al. 2024; Sun et al. 2024; Reda et al. 2023).

In addition to its role in TMD, studies have shown a potential association between higher OBC‐21 scores and increased levels of anxiety, depression, and psychological stress scores (Xu et al. 2021; Medeiros et al. 2020; Cao et al. 2023; Donnarumma et al. 2018). These findings suggest that the impact of oral habits extends beyond the physical domain, implying the presence of a broad association between oral health and psychological well‐being.

When focusing on a specific oral behavior, such as unilateral chewing, a common masticatory pattern where individuals predominantly chew on one side (Han et al. 2024), limited research has examined its relationship with psychological conditions. Unilateral chewing has been found to be one of the most prevalent deleterious oral behaviors, according to studies conducted in Italy and China (63.3% and 47.5%, respectively) (Xu et al. 2021; Reda et al. 2023). A recent study established a correlation between mental stress levels and unilateral chewing but found no significant association between anxiety levels and unilateral chewing habits when assessed by the Perceived Stress Scale (PSS) and Hamilton Anxiety Rating Scale (HAM‐A) (Bano et al. 2024).

Despite its high prevalence, no studies have specifically investigated the relationship between unilateral chewing and anxiety, as assessed by the Generalized Anxiety Disorder‐7 (GAD‐7) scale, or its association with somatic pain severity, measured by the Patient Health Questionnaire (PHQ‐15). Examining item‐level responses within the OBC‐21 in relation to GAD‐7 and PHQ‐15 allows identification of oral behaviors closely linked to psychological conditions, providing insight for research and clinical assessment. Both GAD‐7 and PHQ‐15 are well‐established, validated self‐report tools for assessing anxiety and somatic pain disorders, respectively (Löwe et al. 2008; Kroenke et al. 2002). Given their exposure to transitional life stressors, which may affect both psychological well‐being and oral habits (Cao et al. 2023; Chen et al. 2024; Câmara‐Souza et al. 2023; Manfredini and Lobbezoo 2009; Saracutu et al. 2024a, 2024b), university students represent a relevant population for investigating such a relationship.

Thus, the aim of this study was to assess the prevalence of unilateral chewing behavior, anxiety, and somatic symptoms among university students in Italy and to investigate the association between such behavior and sex, anxiety score, and somatic pain severity.

## 2 Materials and Methods

### 2.1 Study Design and Population

A cross‐sectional study was conducted between January and June 2021 to assess the prevalence of unilateral chewing patterns and their relation with sex, GAD‐7, and PHQ‐15. A convenience sample of students from “Università degli Studi di Trieste” in Italy was recruited, representing various majors, academic levels (undergraduate and postgraduate), and nationalities. Eligibility for participation was open to all registered students, with exclusions made for incomplete survey submissions to ensure data integrity.

### 2.2 Ethical Consideration

Prior to the initiation of the study, ethical approval was obtained for the study protocol. The protocol, which detailed the study design, methodologies, participant recruitment procedures, data collection process, and specific objectives, was carefully reviewed and submitted to the Research and Ethics Committee (REC) of the Ateneo Units and was granted approval (IRB ID: n. 89/11062018).

### 2.3 Sample Size Calculation

The sample size was calculated using Epi Info 7. In the absence of similar studies on the Italian population, the calculation was based on the single population proportion formula. This approach utilized a 50% proportion (which is the most conservative estimate for maximum sample size), a 95% confidence interval (CI), and a 5% margin of error. The total population size of the study was 17,830 students, which was factored into the calculation to ensure the sample size was adequate to achieve reliable and statistically significant results. The minimum final sample size required was 377 participants.

$$n=\frac{Z^{2}\times P\;(1-P)}{e^{2}}÷\left[1+\frac{Z^{2}\times P\;\left(1-P\right)}{e^{2}\times N}\right]=\frac{1.96^{2}\times 0.5(1-0.5)}{0.05^{2}}÷\left[1+\frac{1.96^{2}\times 0.5\left(1-0.5\right)}{0.05^{2}\times 17,830}\right]=377,$$

where: *n* = required sample size, *z* = 1.96 (*z*‐score for 95% confidence level), *p* = 0.5 (estimated population proportion), *e* = 0.05 (margin of error), and *N* = 17,830 (population size).

To enhance the power and generalizability of the study, the sample size was further increased and included a total of 1536 university students.

### 2.4 Data Collection Methods

The online questionnaire was distributed to the entire University population via institutional emails sent from the administration office. All participants provided informed consent in accordance with ethical guidelines. To prevent duplicate responses, each participant was allowed to access and submit the survey only once, and the overall response rate was monitored throughout the study.

The survey first identified participants' age and sex before moving to the main sections of the questionnaire.

#### 2.4.1 Unilateral Chewing Pattern

Participants were asked to report the frequency of chewing food on one side. Responses were based on the OBC‐21 (Markiewicz et al. 2006; Ohrbach et al. 2008), with the following options: None of the time, a little of the time, some of the time, most of the time, and all of the time.

#### 2.4.2 GAD

Anxiety was assessed using the validated Italian version of the self‐report GAD‐7 (Spitzer et al. 2006; Bolgeo et al. 2023). The GAD‐7 consists of 7 questions, each with four possible responses: “Not at all” (score: 0), “Several days” (score: 1), “More than half of the days” (score: 2), and “Nearly every day” (score: 3). A cumulative score was calculated by summing the individual responses. Participants with a cumulative GAD‐7 score ≥ 10 were considered to have anxiety.

#### 2.4.3 Somatic Symptom Severity

Italian version of the self‐report PHQ‐15 was used to assess the severity of somatic symptoms (Kroenke et al. 2002; Interian et al. 2006; Ohrbach 2014). This 15‐item questionnaire asked participants to rate the degree to which various symptoms bothered them, using a 3‐point Likert scale: “Not bothered at all” (score: 0), “Bothered a little” (score: 1), and “Bothered a lot” (score: 2). The total score was obtained by summing the individual responses. Participants with a PHQ score ≥ 10 were considered to have moderate‐severe somatic symptoms.

### 2.5 Statistical Analysis

The data were analyzed using SPSS version 26. Descriptive statistics were applied to summarize the characteristics of the study participants, as well as the prevalence of unilateral chewing behavior, anxiety, and moderate‐severe somatic symptoms, presented in terms of percentages and frequencies. To evaluate the association between sex and unilateral chewing behavior, the Pearson Chi‐square test was performed, followed by Cramér's V test to quantify the strength of the association. To examine the association between the unilateral chewing pattern, GAD‐7 total scores, and PHQ‐15 total scores, homogeneity of variances was tested using Levene's test. For GAD‐7 total scores, the assumption of variances was violated (*p* = 0.014). Therefore, Welch's ANOVA and Games‐Howell post hoc tests were applied. For PHQ‐15 total scores, variances were equal across groups (*p* = 0.080), so standard ANOVA with Tukey's HSD post hoc tests were used. Subsequently, Ordinal logistic regression was performed to assess the relationship between unilateral chewing behavior, total anxiety score, and total somatic symptom score, controlling for age and sex as potential confounders. A *p*‐value of < 0.05 was considered statistically significant for all analyses.

## 3 Results

A total of 1536 university students participated in this study, with a mean age of 24.7 years (SD ± 6). Seventy‐four percent of the study participants were females, and 26% were males. To determine the prevalence of unilateral chewing behavior, anxiety, and somatic symptoms among female and male populations, the variables were dichotomized. Unilateral chewing behavior was dichotomized in line with a previous study (Xu et al. 2021), with participants reporting the behavior “most” or “all of the time” considered as exhibiting unilateral chewing. Overall, 19% of participants displayed unilateral chewing behavior, with 21% of females and 14% of males affected. Anxiety, defined as a GAD‐7 score ≥ 10, was present in 45% of participants (50% of females and 31% of males), while moderate‐to‐severe somatic symptoms, defined as a PHQ‐15 score ≥ 10, were observed in 45% of the total sample (53% of females and 22% of males). These conditions were assessed independently, and identical prevalence does not imply co‐occurrence within the same individuals. Detailed prevalence data are presented in Table 1.

**Table 1:** Prevalence of unilateral chewing behavior, anxiety, and somatic symptoms among female and male participants.

	Females (%)	Males (%)	Total (%)
	*n* = 1136	*n* = 400	*n* = 1536
Unilateral chewing behavior	242 (21.3%)	55 (13.8%)	297 (19.3%)
Anxiety	574 (50.5%)	126 (31.5%)	700 (45.5%)
Somatic symptoms	607 (53.4%)	89 (22.3%)	696 (45.3%)

*Note: n*, number of participants.

### 3.1 Factors Associated With Unilateral Chewing Behavior

All analyses were conducted using the full frequency categories of unilateral chewing behavior from the OBC‐21, alongside total scores of GAD‐7 and PHQ‐15, to preserve the full variability of the data.

Pearson Chi‐square test revealed a significant association between sex and unilateral chewing pattern (*p* < 0.001). However, Cramér's V test indicated that the strength of this association is weak to moderate, with a value of 0.125.

**Figure 1:**
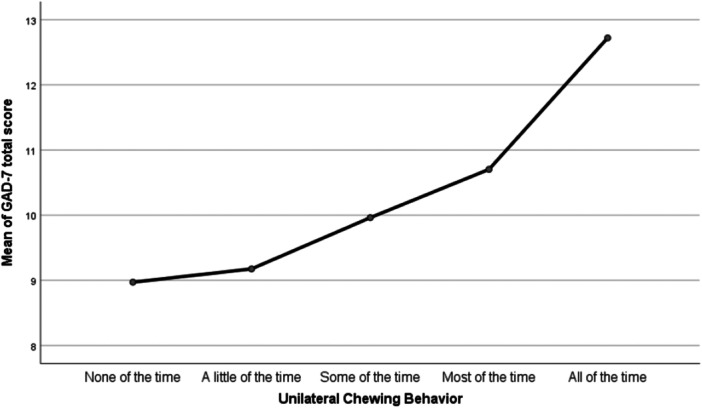
Means plot showing the relationship between total Anxiety Scores (GAD‐7) and frequency of unilateral chewing behavior.

Welch's ANOVA implied the presence of a significant relationship between total GAD‐7 scores and unilateral chewing behavior categories (*p* < 0.001). Post hoc analysis using the Games‐Howell test further quantified this relationship. Participants who reported unilateral chewing behavior all of the time showed approximately four‐point higher total GAD‐7 scores than those who either did not engage in the behavior “none of the time” or did so only “a little of the time.” Similarly, participants who reported unilateral chewing behavior “all of the time” had anxiety scores three points higher than those who exhibited the behavior “some of the time.” No significant difference was reported between participants who reported unilateral chewing behavior all of the time and most of the time. These findings are presented in Table 2, with the corresponding means plot shown in Figure 1.

**Table 2:** Post hoc analysis across chewing behavior groups.

Dependent variable	Independent variable		Mean	*p* value
GAD‐7 total scores	**Unilateral chewing behavior**			
	All of the time	None of the time	3.74	< 0.001
		A little of the time	3.54	< 0.001
		Some of the time	2.75	0.002
		Most of the time	2.01	0.059
PHQ‐15 total scores	**Unilateral chewing behavior**			
	All of the time	None of the time	3.20	< 0.001
		A little of the time	2.44	< 0.001
		Some of the time	1.35	0.135
		Most of the time	1.18	0.293

*Note:* Post hoc tests were selected based on homogeneity of variances. Games‐Howell post hoc test was used for GAD‐7 total scores, and Tukey HSD post hoc test was used for PHQ‐15 total scores.

Abbreviations: GAD‐7, Generalized Anxiety Disorder Assessment Score; PHQ‐15, Patient Health Questionnaire.

**Figure 2:**
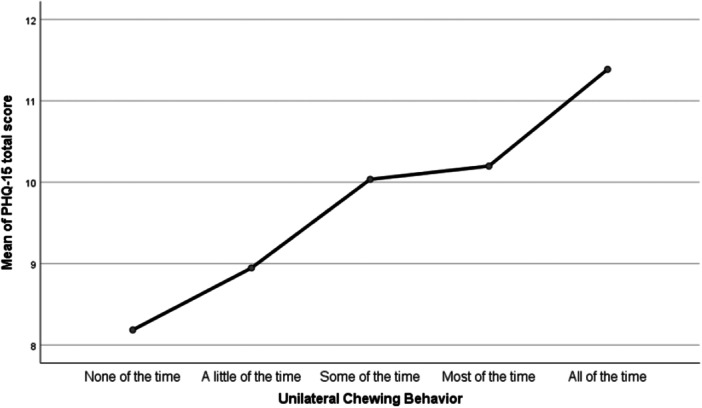
Means plot showing the relationship between total Somatic Symptom Severity Scores (PHQ‐15) and frequency of unilateral chewing behavior.

Moreover, a significant association (*p* < 0.001) was reported when performing the one‐way ANOVA to assess the presence of a relationship between total PHQ‐15 scores and unilateral chewing behavior. Post hoc analysis using Tukey's HSD test indicated no significant difference in somatic symptom severity scores (PHQ‐15) between participants who reported unilateral chewing “all of the time,” “most of the time,” or “some of the time.” However, participants who reported unilateral chewing “all of the time” had somatic symptom scores that were three points higher than those who never engaged in unilateral chewing and two points higher than those who engaged in the behavior “a little of the time.” Detailed results are presented in Table 2, with the corresponding mean plot shown in Figure 2.

Ordinal Regression analysis showed a significant association between unilateral chewing behavior and total anxiety score (GAD‐7 total score). Specifically, each one‐point increase in GAD‐7 score was associated with a 2.2% increase in the odds of being in a higher category of unilateral chewing (OR = 1.022, 95% CI [0.001, 0.044], *p* = 0.044). Similarly, the total somatic symptom severity score (PHQ‐15) was associated with unilateral chewing frequency. Each one‐point increase in the PHQ‐15 total score was associated with a 5.5% increase in the odds of being in a higher category of unilateral chewing, that is, engaging more frequently in the unilateral chewing behavior (OR = 1.055, 95% CI [0.028, 0.075], *p* < 0.001). Although both associations were statistically significant, the effect sizes were small, and neither age nor sex showed a significant association with unilateral chewing frequency.

## 4 Discussion

This study was employed to investigate the relationship between anxiety, somatic symptoms, and unilateral chewing behavior. For this, the validated GAD‐7 and PHQ‐15 scores were used.

Several studies have highlighted the prevalence of unilateral chewing behaviors across different populations (Christensen and Radue 1985; Mc Donnell et al. 2004; Zamanlu et al. 2012) and have reported that this behavior may be associated with anterior disc displacement in the temporomandibular joint, particularly when chewing hard food, which could contribute to the development of temporomandibular disorders (Ratnasari et al. 2011; La Touche et al. 2019).

While much of the existing research has focused on the association between overall oral behaviors and psychological conditions (Vrbanović et al. 2022; Marpaung et al. 2024), especially in TMD patients, no studies have specifically examined the relationship between unilateral chewing behavior, GAD‐7, and PHQ‐15 scores.

The study results found that 19% of the participants exhibited unilateral chewing behavior “most of the time” and “all of the time”, with a significant mild to moderate association between sex and unilateral chewing behavior, where females reported more frequent engagement in this behavior compared to males. Although studies have shown higher unilateral prevalence rates, those studies were focusing on patients with TMD, making those high rates reasonable (Xu et al. 2021; Reda et al. 2023; Leketas et al. 2017).

Regarding anxiety, the study found a 45% prevalence rate among university students in Italy. A systematic review reported an average prevalence of anxiety of 39.65% among university students (Ahmed et al. 2023), 84% among Pakistani university students (Asif et al. 2020), and a comparable rate in Turkey (Sakin Ozen et al. 2010). Additionally, females showed significantly higher anxiety levels, which comes in conformity with other studies (McLean et al. 2011). These findings underscore the importance of considering the broader health impacts of anxiety, including its effect on oral health.

Somatic symptoms were also prevalent among the study participants, with 45% reporting moderate to severe symptoms, aligning with findings from other studies that assessed somatic symptom severity using the PHQ‐15. A cross‐sectional study revealed that the prevalence of somatic symptoms in Slovakia ranged between 69% and 72%, where somatic complaints were the most reported mental health problem during the COVID‐19 pandemic (Gavurova et al. 2022). This high prevalence necessitates the need to address the various facets of this widespread mental health issue. It is important to mention that sex has a significant role in the development of somatic pain symptoms. Our study results revealed that females showed higher total somatic symptom scores than males. Such results have been validated by parallel results from other studies (Kocalevent et al. 2013).

Accordingly, this study was able to highlight a significant relationship between unilateral chewing behavior and anxiety, even after controlling for age and sex as possible confounders. Participants with higher anxiety scores exhibited more frequent unilateral chewing behaviors than those with lower anxiety levels. Previous studies have identified a link between psychological stress and mastication frequency (Kubo et al. 2015; Petrowski et al. 2014), as well as a correlation between total OBC‐21 scores and anxiety (Xu et al. 2021; Medeiros et al. 2020; Cao et al. 2023). A single recent study explored the relationship between stress and unilateral chewing patterns and was able to establish a correlation between mental stress levels and unilateral chewing but found no significant association between anxiety levels and unilateral chewing habits when assessed by the PSS and HAM‐A (Bano et al. 2024). From this point comes the novelty of this research in assessing the relationship between GAD‐7 scores and unilateral chewing behavior frequency.

Similarly, the study identified the relationship between total somatic symptom severity scores and unilateral chewing after controlling for age and sex. Participants with more frequent unilateral chewing behavior exhibited higher somatic symptom scores than those who chewed unilaterally less often, making this study the first to address this aspect. A previous study highlighted a potential association between the presence of somatic symptoms and oral behaviors, as measured by the OBC‐21 among patients with TMD (Yap et al. 2025). Building on this, our findings indicate a further possible association between a single oral behavior, unilateral chewing, and the severity of somatic symptoms, independent of TMD status.

The findings from a study assessing the effect of singing on pain and psychological well‐being, and this study present divergent perspectives on behaviors listed in the OBC‐21, particularly with respect to singing and unilateral chewing (van Selms et al. 2022). While singing was categorized as a positive oral behavior that can alleviate pain and improve psychological well‐being in patients with TMD, unilateral chewing behavior was associated with heightened anxiety and somatic symptoms severity, suggesting the complex nature of oral behaviors and their impact on health (van Selms et al. 2022).

This study was the first to assess the relationship between unilateral chewing behavior with respect to total anxiety scores and total somatic symptom severity scores. Additionally, the study included 1536 participants, where the sample size needed was 377, which gives more power to the study. Moreover, the use of the Italian‐translated and validated GAD‐7 and PHQ‐15 scores further strengthens the reliability of this study. Although age and sex can be considered possible confounding factors, after adjusting for age and sex in the regression model, the relationship between unilateral chewing behavior, total anxiety score, and total somatic symptom score remained significant, highlighting that unilateral chewing behavior is independently associated with both anxiety and somatic symptoms, regardless of age and sex. Previous studies indicated that emotional or uncontrolled eating is associated with elevated anxiety symptoms (Cifuentes et al. 2022), whereas mindful eating correlates with lower psychological symptomatology (Verrier and Day 2022). Based on these findings, a possible mechanism is that individuals with higher psychological symptoms may adopt rapid or emotionally driven eating behaviors, promoting habitual unilateral mastication, while individuals engaging in mindful eating may exhibit slower, bilaterally balanced mastication. Finally, the novelty of this study highlights the potential value of interprofessional collaboration between dental and psychological healthcare providers to support patients' masticatory performance and overall oral health, alongside psychological well‐being. Understanding the observed association between unilateral chewing and psychological distress suggests that comprehensive assessments could, in the future, include evaluations for unilateral chewing habits as part of dental rehabilitation plans. Such assessments may help guide targeted educational interventions that might focus on the importance of balanced mastication and avoiding overuse of one side of the jaw, alongside techniques such as biofeedback and cognitive‐behavioral therapy to reduce parafunctional habits. However, given the modest effect sizes observed, reliance on self‐reported data, and lack of evidence to support the association, these applications remain hypothetical and warrant further research before being implemented as a clinical recommendation.

On the other hand, some limitations should be noted. The study could have benefited from further categorization of participants based on socio‐demographic, health status (presence of TMD), and academic discipline, which would have provided a more insightful understanding of the characteristics of those with high scores and unilateral chewing behavior. Factors such as muscle or articular pain, dental pain, oral lesions, dental sensitivity, tooth loss, fractured teeth, or deficient restorations were not assessed; these conditions could influence unilateral chewing behavior in the timeframes considered by the questionnaires and may introduce a form of misclassification or selection bias. Although the total number of responses exceeded the calculated sample size, the low response rate of 8.6% suggests the possibility of selection bias. Additionally, self‐selection bias may be present, as participants who chose to respond could differ from non‐respondents, potentially affecting the generalizability of the results. Information bias exists due to the online questionnaire format. While clear language and instructions were provided via email, the absence of an interviewer may have led to some misunderstandings. The study results are limited to university students, which hinders their applicability to the general population. Furthermore, the cross‐sectional design of this study limits the ability to determine the direction or causality of the associations observed between unilateral chewing behavior, anxiety, and somatic symptoms. It should be noted that the study was conducted during the COVID‐19 pandemic, which may have contributed to higher levels of anxiety and somatic symptoms, and this should be considered when comparing our findings with pre‐ or post‐pandemic data. Finally, pandemic‐related constraints prevented assessment of factors such as missing teeth, denture fit, or tooth pain, which may have introduced residual confounding. Future studies are recommended to address these limitations.

## 5 Conclusion

The study findings indicate a significant association between unilateral chewing behavior, anxiety, and somatic symptom severity scores, with a modest strength as reflected by the effect sizes. These results suggest a potential link between this oral behavior and psychological distress; however, causal relationships cannot be inferred. Further research is needed to clarify the mechanisms underlying this association and its potential relevance for clinical assessment.

## Author Contributions

Concept and design aspects were led by Bachar Reda and Mariam Hmeidan. Data acquisition and statistical analysis were conducted by Bachar Reda and Mariam Hmeidan. Analysis and interpretation of the data were performed collectively by Bachar Reda, Luca Contardo, and Mariam Hmeidan. Mariam Hmeidan was responsible for drafting the manuscript, which was critically revised for important intellectual content by Bachar Reda and Luca Contardo. All authors reviewed and approved the final manuscript.

## Funding

The authors have nothing to report.

## Ethics Statement

Prior to the initiation of the study, ethical approval was obtained for the study protocol. The protocol, which detailed the study design, methodologies, participant recruitment procedures, data collection process, and specific objectives, was carefully reviewed and submitted to the Research and Ethics Committee (REC) of the Ateneo Units and was granted approval (IRB ID: n. 89/11062018).

## Consent

The authors have nothing to report.

## Conflicts of Interest

The authors declare no conflicts of interest.

## Data Availability

The data that support the findings of this study are available from the corresponding author upon reasonable request.
